# Protocol for a multicenter, real-world study of HIV pre-exposure prophylaxis among men who have sex with men in China (*CROPrEP*)

**DOI:** 10.1186/s12879-019-4355-y

**Published:** 2019-08-15

**Authors:** Hongyi Wang, Yonghui Zhang, Zhu Mei, Yueru Jia, Sequoia I. Leuba, Jing Zhang, Zhenxing Chu, Haibo Ding, Yongjun Jiang, Wenqing Geng, Hong Shang, Junjie Xu

**Affiliations:** 1 NHC Key Laboratory of AIDS Immunology (China Medical University), Department of Laboratory Medicine, The First Affiliated Hospital of China Medical University, No. 155, Nanjingbei Street, Heping District, Shenyang, 110001 Liaoning Province China; 2 Key Laboratory of AIDS Immunology of Liaoning Province, The First Affiliated Hospital of China Medical University, Shenyang, 110001 China; 3 Key Laboratory of AIDS Immunology, Chinese Academy of Medical Sciences, Shenyang, 110001 China; 4 Collaborative Innovation Center for Diagnosis and Treatment of Infectious Diseases, 79 Qingchun Street, Hangzhou, 310003 China; 5 Department of Epidemiology, University of North Carolina at Chapel Hill, Chapel Hill, NC USA

**Keywords:** Pre-exposure prophylaxis, PrEP, HIV, MSM

## Abstract

**Background:**

Pre-exposure prophylaxis (PrEP) is a promising and effective tool to prevent human immunodeficiency virus (HIV) transmission; however, context-specific data to guide optimal implementation are currently lacking in China. This study aims to systematically collect comprehensive, empirical data to determine effective ways to implement PrEP among at-risk men who have sex with men (MSM) in China.

**Methods:**

The *CROPrEP* project, a real-world study of PrEP use, will recruit 1000 high-risk HIV-negative MSM participants from four cities in China, who will be able to choose between daily or event-driven dosing regimens, according to their preference. Participants will be followed up at months 1, 3, 6, 9, and 12 for PrEP provision, clinical evaluation, laboratory testing (e.g., emtricitabine/tenofovir disoproxil fumarate (FTC/TDF) concentrations, and HIV/sexually transmitted infections), alongside detailed, self-administered online questionnaires regarding sexual behaviors, adherence, and attitudes. Online weekly notes will be used to record pill use and sexual practice. Various measurements will be triangulated to assess adherence, including: self-reported adherence, pill count, and drug concentration. A propensity score matching model will be fitted to examine the effectiveness of PrEP use in HIV seroconversion compared with non-PrEP users selected from a local expanding cohort study of HIV-1-negative MSM at participating research centers. Analyses using a generalized estimating equation model will focus on elucidation of the cascade of PrEP implementation, effectiveness, safety, and possible effects of PrEP use on sexual behaviors. This study will provide a comprehensive assessment of real-world PrEP use among Chinese MSM, to develop guidelines and strategies for PrEP implementation in China.

**Discussion:**

The *CROPrEP* project is the first study of the TDF/FTC combination as PrEP in China, which will provide primary data on PrEP implementation, including: the cascade of PrEP use, “real-world” effectiveness, adherence, and safety. The findings from this study have potential to be vital for promoting the integration of PrEP within the portfolio of HIV prevention interventions and developing guidance on PrEP implementation in China.

**Trial registration:**

ChiCTR-IIN-17013762 (Chinese Clinical Trial Registry). Date of registration: 8 December 2017.

## Background

In China, men who have sex with men (MSM) account for more than a quarter of overall new HIV infections [1] and this proportion has doubled in the last decade [2]; hence, MSM represent a key population with an increasing trend of HIV incidence rate over time [3]. According to national Sentinel Surveillance data, the prevalence of HIV among MSM has surged from 1.5% in 2005 to 8.0% in 2015 [4]. Further, a survey in seven cities reported that urban MSM had a higher HIV prevalence of 9.9% [5], with levels as high as 20% in some areas [6]. In the face of such challenges, it is necessary to actively explore novel prevention strategies to effectively control the HIV epidemic among MSM in China.

Pre-exposure prophylaxis (PrEP) refers to a promising prevention strategy, where people uninfected with HIV use antiretroviral drugs to prevent acquisition of the virus, and has been recommended by the World Health Organization and Centers for Disease Control and Prevention (CDC) in the United States for HIV prevention [7–9]. The coformulation of 200 mg emtricitabine (FTC) and 300 mg tenofovir disoproxil fumarate (TDF) (TDF/FTC, Truvada®) is the only drug approved by the US Food and Drug Administration for pre-exposure prophylaxis. Several clinical trials and some real-world studies have shown that daily or event-driven TDF/FTC is effective in preventing HIV in key populations, particularly MSM, when taken correctly [10–15] and more than 40 countries have approved the use of PrEP as a preventive measure [16]. Despite the continued expansion of the PrEP strategy, there are socio-cultural contexts and healthcare infrastructure gaps between low- and middle-income countries (including China) and developed countries, which represent particular challenges for the former in the implementation of PrEP, necessitating a cautious approach [17].

Public health authorities in China have not yet integrated TDF/FTC use for PrEP into packages of HIV prevention interventions because first-hand, high-quality evidence supporting the feasibility of its implementation on the mainland is lacking. Previous cross-sectional studies on the acceptability of PrEP among Chinese MSM reported moderate acceptability of PrEP use (40–90%) [18–21], whereas poor quality evidence suggested that there may be a severe gap between willingness and actual uptake of PrEP among MSM, since a trial in Shanghai found that only 3% of participants actually took daily oral TDF as PrEP [22]. Whether such a huge gap would also exist when daily and event-driven TDF/FTC are promoted, and the factors influencing the implementation of PrEP among MSM in China, are also unknown. To our knowledge, no studies have investigated the feasibility of oral TDF/FTC (i.e., the efficacy, adherence retention, and risk compensation) for HIV prevention among MSM in China. A limited number of trials of daily TDF have suggested that compliance with daily PrEP in MSM is not high [23, 24]; however, there remains a dearth of data reflecting the “real world” feasibility of PrEP use, based on the development of appropriate approaches to PrEP implementation, which may hinder the advance of any PrEP strategy in mainland China.

To address this gap in knowledge, the Mega-projects of National Science Research for the 13th Five­Year Plan financially supported the establishment of the *CROPrEP* project, a PrEP implementation study among Chinese MSM at high risk of acquiring HIV. The project aims to comprehensively assess the feasibility of PrEP use and provide the necessary evidence to support the development of PrEP implementation guidelines in China.

### Research objectives

The objectives of the *CROPrEP* project are to assess among HIV-negative Chinese MSM:

1. The real-life effectiveness of daily or event-driven PrEP use
2. Adherence to PrEP
3. Safety and tolerability
4. The cascade of PrEP and motivations and attitudes toward PrEP use
5. The potential effects of PrEP use on sexual behaviors and incidence of sexually transmitted infections (STIs)

## Methods/design

### Overview of study design

**Fig. 1:**
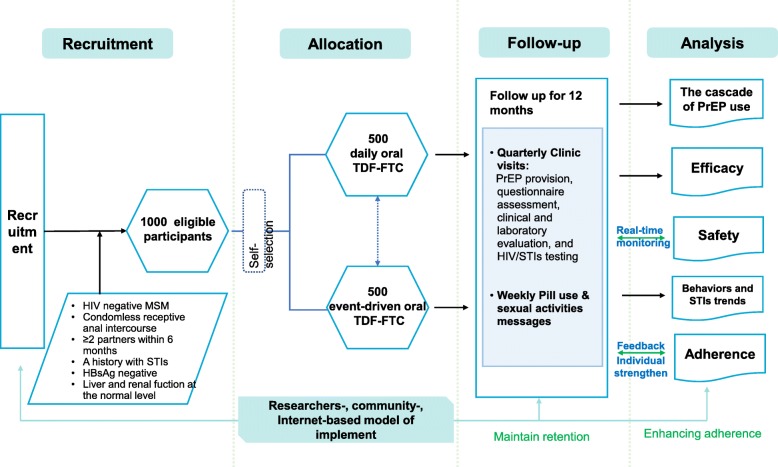
Flow chart of study design

As shown in Fig. 1, the *CROPrEP* project is a multicenter, real-world, prospective cohort study with two arms of daily or event-driven TDF/FTC as PrEP among HIV-negative MSM at high risk of HIV infection in China. This study is being conducted in four metropolitan Chinese cities, which have moderate to high prevalence of HIV among MSM: Shenyang, Beijing, Shenzhen, and Chongqing. In total, 1000 eligible participants will be permitted to choose between daily or event-driven PrEP, based on their preference. Participants will be prospectively followed for 12 months, with quarterly clinic visits, and online weekly notes. The PrEP intervention, in combination with a package of prevention services, will be provided to participants (e.g., free adherence supporting reminders and counseling, sexual health education, panel management by peer counselors, and referrals to relevant clinics for individuals who test positive for either HIV or syphilis). These four study centers have also established an expanding cohort study of 3200 HIV-1-negative MSM for epidemic surveillance and estimation, which can be considered a natural background control for PrEP users, to evaluate the efficacy of PrEP use.

### Sample size

Given the well-documented protective effect of PrEP, the *CROPrEP* project is considered a demonstration project, and the sample size is not determined by power calculations. Based on preliminary survey data of acceptance among MSM in China, the sample size was increased as far as possible, on the basis of fully considering the population of each research center, and a largest sample size of 1000 chosen (Table 1).

**Table 1:** Number of participants recruited by each site

Sites	Event-driven dosing group	Daily dosing group
Shenyang	185	185
Beijing	205	215
Shenzhen	50	40
Chongqing	60	60
Total	500	500

### Project promotion and recruitment of participants

Participant recruitment is performed by both online and offline methods. Online methods rolled out through accounts managed by each research center include advertising on public social platforms, such as Weibo and WeChat. Offline recruitment methods include the following: (1) clinic-based sampling: each study center collaborates with the local CDC and clinic staff will recruit MSM who already attend voluntary counseling and testing clinics, are at high risk of HIV infection, and are willing to participate in the *CROPrEP* after learning about the study; (2) community-based sampling: each study center collaborates with local MSM community-based organizations (CBOs) to recruit MSM in the community; (3) venue-based sampling: eligible MSM will be recruited from suitable venues (e.g., MSM parks, bars, clubs, and bathhouses) through outreach activities; and (4) peer referrals: potential participants will be encouraged to recruit their friends who are MSM or their male partners to participate in this study. All candidates will be preregistered and then invited for a screening visit at the local study center on the principle of competitive enrollment. Prior to the eligibility assessment for the study, written informed consent will be obtained from all participants.

### Participant eligibility and enrollment

As shown in Table 2, the inclusion and exclusion criteria will be used to assess the eligibility of participants in the *CROPrEP* project. These criteria are established by an expert group of researchers, clinicians, and CBO representatives, based on international guidelines on PrEP and the behavior characteristics of MSM in China [3].

**Table 2:** Inclusion and exclusion criteria for participants in the *CROPrEP* project

Inclusion criteria	
• Aged 18–65 years old	
• Test results demonstrate HIV negative	
• Behavioral eligibility criteria	
Participants of male sex at birth and who have sex with men, reporting at least one criterion associated with high risk for HIV infection in the 6 months prior to enrolment as follows:	
- Unprotected (condom-less) receptive anal intercourse with male partners	
- More than two male partners (regardless of condom use and HIV serostatus)	
- Reported STI, such as syphilis, HSV-2, gonorrhea, chlamydia, chancroid, or lymphogranuloma venereum	
- Reported a history of post-exposure prophylaxis	
Note: Individuals in a monogamous relationship with an HIV-1 seronegative partner or a virologically suppressed HIV-1+ partner for > 1 year will not be eligible for participation.	
• Through comprehensive physical examination, including routine urine examinations, hepatic and renal function tests, blood glucose and lipids, and BMD; no serious liver or kidney dysfunction and negative for HBs antigen, without serology indicating osteoporosis, and other indicators are normal	
• Able and being willing to sign written informed consent and participate in the study as procedures require	
• Chinese citizens	
Exclusion criteria	
• HIV-1 infected, or having clinical signs or symptoms consistent with acute viral infection	
• Atopic individual or allergic to the ingredients of the experimental drug or ART	
• Having serious chronic disease, including metabolic diseases (such as diabetes), neurological, and psychiatric disorders	
• Weight < 40 kg or > 140 kg	
• Having osteoporosis: aged ≥50 years with BMD T-score ≤ −2.5; aged < 50 years with BMD Z-score ≤ − 2 and fragility fracture	
• Mental health issues which may compromise participant adherence or safety, including memory loss, cognitive impairment, intellectual disability, or communication disorders	
• Currently, or 30 days prior to enrolment, taking interferon, interleukin, or other immunoregulators	
• Currently taking products containing antiretrovirals	
• Participating in another research study related to HIV and antiretroviral therapy or other intervention study	

Eligibility will be assessed for ongoing comprehensive screening at a clinic, including laboratory testing for antibodies and RNA for HIV, syphilis, herpes simplex virus 2 (HSV-2), IgM and IgG antibodies, hepatitis B virus surface antigen (HBsAg), bone mineral density (BMD), renal and liver function, clinical evaluation with family or personal medical history, concomitant treatment by an experienced physician, and a self-administered questionnaire, to assess sociodemographic factors, current sexual behavior, substance use, and social and psychological information, on their mobile phones. Confirmed eligible participants will be invited to return to the clinic within a week to choose a regimen and receive a supply of TDF/FTC and detailed instructions for PrEP use.

### Drug regimen and provision

The PrEP used in the *CROPrEP* project is Truvada®; one film-coated pill contains 200 mg of FTC and 300 mg of TDF. Participants will receive free Truvada and can choose between taking PrEP on a daily basis, or according to an event-driven regimen, before and after anal sex. The daily dosing regimen is one Truvada pill every 24 h. The event-driven regimen is two Truvada pills 2–24 h before sexual intercourse (or one pill if the last medication was taken 1 to 6 days ago), a pill every 24 h from the first drug intake during the period of sexual activity, including after the last sexual intercourse, and one final Truvada pill approximately 24 h later. Participants will be permitted to switch their PrEP regimen during the study period.

### Study visits

Each participant will be followed up for 12 months and will experience a total of six clinic visits at centers (including screening (baseline) and follow-up (FU) at 1, 3, 6, 9, and 12 months) (Table 3). The visit window for this study is 7 days. For each clinic visit, adverse events and concomitant medication will be documented and HIV/STI testing and monitoring of biological indices for PrEP-related toxicity performed by experienced physicians. Participants will be instructed to complete an online self-administered questionnaire to assess behaviors, psychological status, adherence, side effects, and attitude towards PrEP use in a separate private interview room. Dry blood spots and blood samples will be collected from participants who report that they have taken PrEP during the follow-up interval for TDF/FTC concentration and HIV/STI testing, alongside other clinical evaluations. Participants will be required to return the leftover TDF/FTC to the clinic in exchange for a refill to cover their needs until the next clinic visit.

**Table 3:** Schedule of the *CROPrEP* project

Procedures	Screening/Enrollment	FU 1 m ± 7d^d^	FU 3 m ± 7d	FU 6 m ± 7d	FU 9 m ± 7d	FU 12 m ± 7d
Informed consent	×					
Self-administrated questionnaire	×	×	×	×	×	×
Interviews						
Relevant medical history	×					
Current/concomitant medication	×	×	×	×	×	×
Adverse events or side effects		×	×	×	×	×
Medication return and allocation	×	×	×	×	×	×
HIV/STIs testing						
HIV screening	×	×	×	×	×	×
HIV Western Blot^a^	×	×	×	×	×	×
HIV RNA Pooling PCR^b^	×	×	×	×	×	×
HIV resistance testing^c^						
HIV-1 viral load^c^						
Syphilis	×	×	×	×	×	×
HSV-2	×	×	×	×	×	×
Safety assessment						
Routine blood tests	×	×	×	×	×	×
Routine urine tests	×	×	×	×	×	×
Liver functions tests	×	×	×	×	×	×
Renal functions tests	×	×	×	×	×	×
Blood glucose and lipids	×	×	×	×	×	×
Bone mineral density	×			×		×
Hepatitis B virus	×			×		×
Adherence lab assessment						
Blood drug level testing		×	×	×	×	×

*Abbreviations*: *FU* Follow-up, *m* Month(s), *d* Day, *HSV-2* Herpes simplex virus 2

^a^Anyone who screens positive for HIV will have the results confirmed by western blotting

^b^Anyone who screens negative for HIV will have the results confirmed by HIV RNA pooling PCR test

^c^Anyone who tests positive for HIV will have their samples further tested for HIV resistance and viral load

^d^Visit window, 7 days

Online weekly notes will be sent to the mobile phones of participants weekly, to feedback their short-term pill use and sexual practices. Participants will be instructed to complete the message every week, to limit recall bias.

During the follow-up period, researchers will benefit from leveraging CBOs and the internet to strengthen group- and individual-level supervision of adherence to medication and cohort management. This multifaceted strategy will include a panel of CBO counselors, providing interactive peer counseling focusing on study retention; a Short Message Service application, providing routine online medication reminders and follow-up visit reminders, along with a live chat; and clinicians and study staff, providing one-on-one personalized compliance support, counseling, and cohort maintenance.

### Laboratory procedures

As summarized in Table 4, during each clinical visit, participants will be asked to provide blood and urine specimens for monitoring of HIV infection, STIs (such as HSV-2 and syphilis), and objective measures for adherence and safety of PrEP use. Analysis of blood TDF/FTC concentrations will be performed in the Key Laboratory of AIDS Immunology of National Health Commission, Department of Laboratory Medicine, The First Affiliated Hospital, China Medical University, in Shenyang, China. All other testing will be conducted by nationally certified laboratories, located at each research center. Dry blood spots and serum samples will be collected at every visit for TDF/FTC drug level testing.

**Table 4:** Laboratory Parameters

Laboratory Parameter	Test
HIV	HIV serostatus is evaluated by ELISA (InTec Products Company, Xiamen, China), and confirmed with an HIV-1/2 western blot (HIV Blot 2.2 WBTM, Genelabs Diagnostics, Singapore). The results of anyone who screens negative for HIV will be confirmed by HIV RNA pooling PCR test (COBAS AmpliPrep /COBAS TaqMan, Roche)
HIV resistance	RNA sequencing
HIV viral load testing	COBAS AmpliPrep /COBAS TaqManv (Roche)
Syphilis	RPR (Shanghai Rongsheng, Shanghai, China) and TPPA (Fujirebio Inc., Tokyo, Japan)
Hepatitis B	HBsAg (Vitros 5600)
HSV-2	IgM-HSV-2, IgG-HSV-2 (Beier Bioengineering, Beijing, China)
Biochemistry	AST, ALT, total bilirubin, creatinine, creatinine clearance, serum phosphate, blood glucose, and lipids (Roche)
Routine blood tests	Full blood count: hemoglobin, leucocytes, platelets; differential count: absolute neutrophil count, absolute lymphocyte count (Mindray BC-5800)
Routine urine tests	Proteinuria (Mindray EH-2080)
Blood drug level testing	LC-MS (AB SCIEX API 6500+)

*Abbreviations*: *ELISA* Enzyme-linked immunosorbent assay, *RPR* Rapid plasma reagin, *TPPA Treponema pallidum* particle agglutination assay, *HSV-2* Herpes simplex virus 2, *HBsAg* Hepatitis B surface antigen, *AST* Aspartate transferase, *ALT* Alanine transferase, *LC-MS* Liquid chromatography-mass spectrometry

If a participant is found to have seroconverted to HIV, his participation in the study will be terminated immediately and he will be called for a final visit. At the final visit, study staff will collect unused pills, conduct resistance and viral load testing, and provide referrals to an HIV clinic for treatment. The study staff will report each case of seroconversion to the sponsor.

### Safety assurance

Safety of TDF/FTC will be monitored by laboratory ratings, based on vital signs and adverse events recorded on a paper case report form at each clinic visit, and the results of causal association and severity assessment will be independently evaluated by two physicians. Any reported adverse events will be clinically tracked until they are restored or stabilized. Serious adverse events are life-threatening events, including HIV seroconversions that must be reported to the sponsor within one business day, and the lead ethics committee within 72 h.

### Data collection and quality assurance

**Fig. 2:**
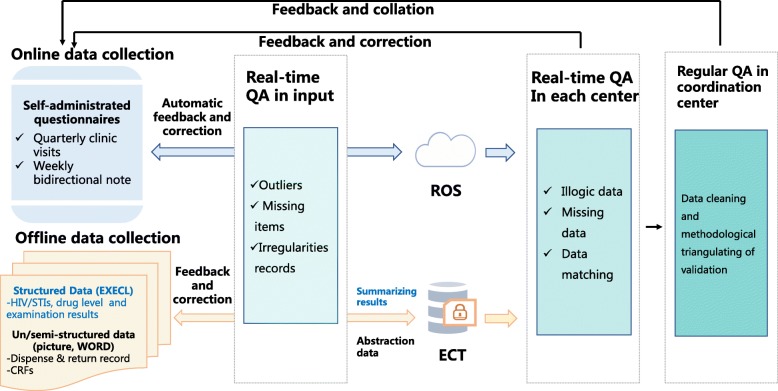
Flowchart of data quality assurance procedures. QA, quality assurance; ROS, real-time online storage; ECT, encrypted compression transmission

The results from both types of data collection will be triangulated to assess various end points. A specialized platform for questionnaire collection will be used to collect self-administered, structured questionnaires to assess socio-demographic information, perceived skills, willingness, impact of PrEP use on behaviors and mental status, self-reported adherence, and side effects. All examination results will be interpreted and organized into structured data by professional physicians. For unstructured data, such as medical records and drug release records, core information on adverse events, medications, and tablet counts will be extracted by trained staff. Over the course of the study, we will establish and strictly operate a system of quality control to ensure the integrity, validity, and authenticity of data (Fig. 2). Besides, data inspection companies are employed as independent from investigators and the sponsor for auditing trial conduct.

### Study end points

As shown in Table 5, primary and secondary end points will be pursued to determine the feasibility and operationalization of PrEP implementation in China.

**Table 5:** End points of the *CROPrEP* project

1. The real-life effectiveness of daily or event-driven PrEP use:	
— HIV incidence among individuals using different PrEP regimens, compared with that of non-PrEP users randomly selected by propensity score from local expanding cohort studies of HIV-negative MSM	
— The rate of viral genotype resistance among individuals who seroconvert to HIV during the study period	
2. Adherence to PrEP:	
— Number, proportion and patterns of prescribed doses taken and missed according to self—reported adherence via online questionnaires and weekly messages, reported pill use, and pill counts	
— Testing of FTC/TDF drug concentration in blood samples	
— Percentage of participants who switch regimens and their reasons	
3. Safety and tolerability:	
— Rate of side effects or adverse events related to PrEP use	
— Rate of adverse events related to discontinuation of PrEP or switching PrEP regimens	
4. PrEP cascade and the motivations and attitudes towards PrEP use:	
— The effectiveness of recruitment, according to the origin of roll-out	
— The cascade of PrEP use: acceptability, initiation, choice of regimen, and retention in the study	
— The knowledge, beliefs, motivations, difficulties, behavior, and expectations of participants, and their knowledge and communication regarding their PrEP use	
— Attitudes towards PrEP use and medical care providers	
5. The potential effects of PrEP use on sexual behaviors and incidence of sexually transmitted infections (STIs):	
— Potential changes in the number of sexual partners and the numbers of casual or steady partners	
— Potential changes in condom use	
— Potential changes in the incidence of HSV-2 or syphilis	
— Changes in the use of sexual networks	
— Changes in perception of sexual well-being during sexual intercourse	

Adherence to PrEP will be measured from different perspectives: drug concentration testing, self-reported adherence via questionnaires and weekly messages, self-report to clinician, dispensing and return records, and drug level assessment.

### Data analysis

The sociodemographic characteristics of the study population will be analyzed using descriptive statistics (mean/standard deviation, or median/interquartile range) and distributions compared using χ^2^ or Fisher’s exact tests, as appropriate. For propensity scoring, logistic regression analysis will first be applied to analysis of all baseline features, followed by matching, computed after division into quintiles, and nearest neighbor analysis of estimated propensity scores. Sexual behaviors, medication adherence, and risk of HIV seroconversion will be analyzed using generalized estimating equation models. All computations will be conducted using STATA version 14.0 (IBM Corp).

## Discussion

Although global coverage of PrEP is expanding, this strategy has not been advanced in China because of a lack of first-hand data regarding PrEP implementation. The *CROPrEP* project is supported by the Mega Projects of National Science Research of the 13th Five­Year Plan, and the findings from the project could potentially serve a vital role in promoting the integration of PrEP with the portfolio of HIV prevention interventions and in developing guidance on PrEP implementation in China.

To our knowledge, *CROPrEP* is the first comprehensive effort to obtain real-world data on TDF/FTC as PrEP use as an HIV prevention strategy in China. The four centers have simultaneously established local, large-scale HIV-negative MSM cohorts, which form natural background controls for PrEP users, facilitating evaluation of the prophylactic effects of PrEP in a real-world context. The study will be conducted as a systematic effort to collect comprehensive data and to reflect the cascade of PrEP use, effectiveness, adherence, and safety. The results collected from different perspectives will be triangulated to determine important end points, such as adherence, with multiple assessments of self-reported data, pill counts, and drug level testing. A novel model that integrates existing and new approaches to clinical trials will be applied, including research center-, local community-, and internet-led models, to facilitate more efficient promotion, and achieve medication supervision and queue management at the group and individual levels. To improve the validity of the data, all research centers will strictly follow the unique standardized quality control system via an online platform, constructed for data collection and management.

A limitation of *CROPrEP* is that, as the first large-scale study of PrEP in China, we are focusing only on MSM. While addressing the epidemic among Chinese MSM could significantly control the overall HIV epidemic, future research should focus on other high-risk groups, such as female sex workers. Another limitation is that, despite the use of neutral language and self-administered questionnaires, with the aim of promoting honesty and trust between study participants and facilitators, sensitive questions about personal topics will be asked, and thus answers may be affected by social expectation bias. As MSM are a hidden population, this study will adopt a convenience sampling method, and the results should be extrapolated carefully.

## Data Availability

Not applicable. As no datasets were generated or analyzed at this time as part of the current study.

## Abbreviations

BMD: Bone mineral density
CBOs: Community-based organizations
CDC: Centers for Disease Control and Prevention
FTC: Emtricitabine
HBsAg: Hepatitis B virus surface antigen
HIV: Human immunodeficiency virus
HSV-2: Herpes simplex virus 2
MSM: Men who have sex with men
PrEP: Pre-exposure prophylaxis
STIs: Sexually transmitted infections
TDF: Tenofovir disoproxil fumarate

## References

[1] NCAIDS, NCSTD, China CDC (2018). Update on the AIDS/STD epidemic in China and main response in control and prevention in October, 2017. China J AIDS STD.

[2] (SCAWCO) SCAWCO (2010). China 2010 UNGASS country progress report (2008–2009).

[3] Zhang Wei, Xu Jun-Jie, Zou Huachun, Zhang Jing, Wang Ning, Shang Hong (2016). HIV incidence and associated risk factors in men who have sex with men in Mainland China: an updated systematic review and meta-analysis. Sexual Health.

[4] Tang S, Tang W, Meyers K, Chan P, Chen Z, Tucker JD (2016). HIV epidemiology and responses among men who have sex with men and transgender individuals in China: a scoping review. BMC Infect Dis.

[5] Xu JJ, Tang WM, Zou HC, Mahapatra T, Hu QH, Fu GF (2016). High HIV incidence epidemic among men who have sex with men in China: results from a multi-site cross-sectional study. Infect Dis Poverty.

[6] Zeng G, Feng L, Ouyang L, Lu R, Xu P, Wu G (2014). The dynamic trends of HIV prevalence, risks, and prevention among men who have sex with men in Chongqing, China. Biomed Res Int.

[7] WHO. Guideline on when to start antiretroviral therapy and on pre-exposure prophylaxis for HIV. 2015. https://apps.who.int/iris/bitstream/handle/10665/186275/9789241509565_eng.pdf;jsessionid=0C137CF48FF377B40D33AE58489E7B9B?sequence=1. 26598776

[8] CDC. PREEXPOSURE PROPHYLAXIS FOR THE PREVENTION OF HIV INFECTION IN THE UNITED STATES – 2017 UPDATE. https://www.cdc.gov/hiv/pdf/risk/prep/cdc-hiv-prep-guidelines-2017.pdf.

[9] Holmes David (2012). FDA paves the way for pre-exposure HIV prophylaxis. The Lancet.

[10] Molina JM, Charreau I, Spire B, Cotte L, Chas J, Capitant C, et al. Efficacy, safety, and effect on sexual behaviour of on-demand pre-exposure prophylaxis for HIV in men who have sex with men: an observational cohort study. Lancet HIV. 2017;4(9):e402–10. 10.1016/S2352-3018(17)30089-9 28747274

[11] Molina Jean-Michel, Capitant Catherine, Spire Bruno, Pialoux Gilles, Cotte Laurent, Charreau Isabelle, Tremblay Cecile, Le Gall Jean-Marie, Cua Eric, Pasquet Armelle, Raffi François, Pintado Claire, Chidiac Christian, Chas Julie, Charbonneau Pierre, Delaugerre Constance, Suzan-Monti Marie, Loze Benedicte, Fonsart Julien, Peytavin Gilles, Cheret Antoine, Timsit Julie, Girard Gabriel, Lorente Nicolas, Préau Marie, Rooney James F., Wainberg Mark A., Thompson David, Rozenbaum Willy, Doré Veronique, Marchand Lucie, Simon Marie-Christine, Etien Nicolas, Aboulker Jean-Pierre, Meyer Laurence, Delfraissy Jean-François (2015). On-Demand Preexposure Prophylaxis in Men at High Risk for HIV-1 Infection. New England Journal of Medicine.

[12] Grant Robert M, Anderson Peter L, McMahan Vanessa, Liu Albert, Amico K Rivet, Mehrotra Megha, Hosek Sybil, Mosquera Carlos, Casapia Martin, Montoya Orlando, Buchbinder Susan, Veloso Valdilea G, Mayer Kenneth, Chariyalertsak Suwat, Bekker Linda-Gail, Kallas Esper G, Schechter Mauro, Guanira Juan, Bushman Lane, Burns David N, Rooney James F, Glidden David V (2014). Uptake of pre-exposure prophylaxis, sexual practices, and HIV incidence in men and transgender women who have sex with men: a cohort study. The Lancet Infectious Diseases.

[13] Hosek Sybil G., Green Keith R., Siberry George, Lally Michelle, Balthazar Christopher, Serrano Pedro A., Kapogiannis Bill, The Adolescent Medicine Trials Netw (2013). Integrating Behavioral HIV Interventions Into Biomedical Prevention Trials With Youth: Lessons From Chicago's Project PrEPare. Journal of HIV/AIDS & Social Services.

[14] Mutua Gaudensia, Sanders Eduard, Mugo Peter, Anzala Omu, Haberer Jessica E., Bangsberg David, Barin Burc, Rooney James F., Mark David, Chetty Paramesh, Fast Patricia, Priddy Frances H. (2012). Safety and Adherence to Intermittent Pre-Exposure Prophylaxis (PrEP) for HIV-1 in African Men Who Have Sex with Men and Female Sex Workers. PLoS ONE.

[15] Grant RM, Lama JR, Anderson PL, McMahan V, Liu AY, Vargas L, et al. Preexposure chemoprophylaxis for HIV prevention in men who have sex with men. New Engl J Med. 2010;363(27):2587–99. 10.1056/NEJMoa1011205 PMC3079639 21091279

[16] AVAC. Regulatory status of TDF/FTC for PrEP. 2019. https://www.avac.org/infographic/regulatory-status-tdfftc-prep.

[17] Wei Chongyi, Raymond H Fisher (2018). Pre-exposure prophylaxis for men who have sex with men in China: challenges for routine implementation. Journal of the International AIDS Society.

[18] Zhou Feng, Gao Lei, Li Shuming, Li Dongliang, Zhang Lifen, Fan Wensheng, Yang Xueying, Yu Mingrun, Xiao Dong, Yan Li, Zhang Zheng, Shi Wei, Luo Fengji, Ruan Yuhua, Jin Qi (2012). Willingness to Accept HIV Pre-Exposure Prophylaxis among Chinese Men Who Have Sex with Men. PLoS ONE.

[19] Zhang Yan, Peng Bin, She Ying, Liang Hao, Peng Hong-Bin, Qian Han-Zhu, Vermund Sten H., Zhong Xiao-Ni, Huang Ailong (2013). Attitudes Toward HIV Pre-Exposure Prophylaxis Among Men Who Have Sex with Men in Western China. AIDS Patient Care and STDs.

[20] Han Jing, Bouey Jennifer ZH, Wang Liming, Mi Guodong, Chen Zihuang, He Ying, Viviani Tara, Zhang Fujie (2019). PrEP uptake preferences among men who have sex with men in China: results from a National Internet Survey. Journal of the International AIDS Society.

[21] Wang Xia, Bourne Adam, Liu Pulin, Sun Jiangli, Cai Thomas, Mburu Gitau, Cassolato Matteo, Wang Bangyuan, Zhou Wang (2018). Understanding willingness to use oral pre-exposure prophylaxis for HIV prevention among men who have sex with men in China. PLOS ONE.

[22] Ding Yingying, Yan Huamei, Ning Zhen, Cai Xiaofeng, Yang Yin, Pan Rong, Zhou Yanqiu, Zheng Huang, Gao Meiyang, Rou Keming, Wu Zunyou, He Na (2016). Low willingness and actual uptake of pre-exposure prophylaxis for HIV-1 prevention among men who have sex with men in Shanghai, China. BioScience Trends.

[23] Qu Dou, Zhong Xiaoni, Xiao Guiyuan, Dai Jianghong, Liang Hao, Huang Ailong (2018). Adherence to pre-exposure prophylaxis among men who have sex with men: A prospective cohort study. International Journal of Infectious Diseases.

[24] Hu Y, Zhong XN, Peng B, Zhang Y, Liang H, Dai JH, et al. Associations between perceived barriers and benefits of using HIV pre-exposure prophylaxis and medication adherence among men who have sex with men in Western China. BMC Infect Dis. 2018;18(1):575. 10.1186/s12879-018-3497-7 PMC6238290 30442106

